# Dr. Zippie Brooks Wales

**Published:** 1912-08

**Authors:** 


Dr. Zippie Brooks Wales, of Elmira, wife of Dr. T. A. Wales, died July 4, following an operation, performed June 22. She was born in Winhall, Vt., and was a sister of Mrs. Dr. S. O. Gleason who, with her husband, established a well-known sanitarium. She was married to Dr. Wales Sept. 26, 1872. The following year, both graduated in medicine, he at the University of Pennsylvania, she at the Woman's Medical College of Philadelphia. After being associated with the Drs. Gleason in sanitarium work for some years, they engaged in private practice in Elmira. Dr. Zippie Brooks Wales was a prominent member of the Daughters of the American Revolution, of the Humane Society, and of various other philanthropic organizations.

